# Impact of breed and sex on porcine endocrine transcriptome: a bayesian biometrical analysis

**DOI:** 10.1186/1471-2164-10-89

**Published:** 2009-02-24

**Authors:** Miguel Pérez-Enciso, André LJ Ferraz, Ana Ojeda, Manel López-Béjar

**Affiliations:** 1 Departament de Ciència Animal i dels Aliments, Facultat de Veterinària, Universitat Autònoma de Barcelona, 08193 Bellaterra, Spain; 2 Institut Català de Recerca i Estudis Avançats (ICREA), C/Lluis Companys 23, 08010 Barcelona, Spain; 3 Faculdade de Ciências Agrárias e Veterinária, Universidade Estadual Paulista (UNESP), 14884-900 Jaboticabal – SP, Brazil; 4 Departament de Sanitat i d'Anatomia Animals, Facultat de Veterinária, Universitat Autònoma de Barcelona, 08193 Bellaterra, Spain

## Abstract

**Background:**

Transcriptome variability is due to genetic and environmental causes, much like any other complex phenotype. Ascertaining the transcriptome differences between individuals is an important step to understand how selection and genetic drift may affect gene expression. To that end, extant divergent livestock breeds offer an ideal genetic material.

**Results:**

We have analyzed with microarrays five tissues from the endocrine axis (hypothalamus, adenohypophysis, thyroid gland, gonads and fat tissue) of 16 pigs from both sexes pertaining to four extreme breeds (Duroc, Large White, Iberian and a cross with SinoEuropean hybrid line). Using a Bayesian linear model approach, we observed that the largest breed variability corresponded to the male gonads, and was larger than at the remaining tissues, including ovaries. Measurement of sex hormones in peripheral blood at slaughter did not detect any breed-related differences. Not unexpectedly, the gonads were the tissue with the largest number of sex biased genes. There was a strong correlation between sex and breed bias expression, although the most breed biased genes were not the most sex biased genes. A combined analysis of connectivity and differential expression suggested three biological processes as being primarily different between breeds: spermatogenesis, muscle differentiation and several metabolic processes.

**Conclusion:**

These results suggest that differences across breeds in gene expression of the male gonads are larger than in other endocrine tissues in the pig. Nevertheless, the strong presence of breed biased genes in the male gonads cannot be explained solely by changes in spermatogenesis nor by differences in the reproductive tract development.

## Background

It is well known that variability at the transcriptome is in part due to genetic causes, much like any other complex phenotype, e.g., [1]. Thus, the large phenotypic differences that we observe between extant breeds of domestic species or between different ecotypes in wild species must be correlated also to differences at the transcriptome level. The extent of these differences and their nature is, however, not fully known. Considering, moreover, that the transcriptome programme differs widely between tissues and across development stages of the same organism while simultaneously many expression levels are highly inter correlated adds an additional layer of complexity to the problem.

Although the differences between tissues and across development stage transcriptomes is a well known fact [2-4], the variability across tissues or development stages is less studied than within a single tissue due to increased costs of longitudinal or across-tissue studies. Certainly, choosing the tissue and the timing to be studied is a critical aspect of any experimental design in transcriptomics. Yet, this choice is not necessarily obvious. An example is growth and leanness in livestock. These two traits are the most important selection objectives in the majority of breeding programmes. But they are very complex phenotypes that depend on a large number of tissues. As for growth, the most valuable tissue in economic terms is muscle. Although muscle would be a logical tissue to be studied, muscle development does depend on signals external to the muscle itself like endocrine and paracrine factors that also change along development. Similarly, differences in the amount of fat tissue are more likely to be caused by genetic signals that originate in the hypothalamus or in endocrine tissues rather than in the fat tissue itself.

In order to study the impact of breed differentiation on the pig's transcriptome, we have analyzed the breed and sex differences across different tissues. Among tissues that are of interest, those involved in the different endocrine axes stand out as a promising choice, considering their fundamental biological role and that their transcriptomes have not been widely analyzed. Here we report a detailed microarray analysis of five tissues that make up two main endocrine axes, the HPTA (hypothalamic-pituitary-gonadal) and HPT (hypothalamic-pituitary-thyroid) axes, plus fat tissue, in four highly divergent porcine breeds. Both HPTA and HPT axes are highly influential endocrine axes and, we conjectured, must be responsible for at least some of the large phenotypic differences between breeds caused by artificial selection in livestock, e.g., in fat content.

## Results

### General overview

The tissues sampled were hypothalamus (HYPO), adenohypophysis (AHYP), thyroid gland (THYG), gonads (GONA) from both sexes, males (GONAM) and females (GONAF), and back fat tissue (FATB). Some of their primary endocrine roles are in Additional file 1. The four breeds were Large White (LW), Duroc (DU), Youli (YL) and Iberian (IB). The Large White is a very lean and high growth breed, it is used as sire (male) line. Although there are many different Duroc lines differing in their lean content and growth performance, the Duroc line employed here was a maternal line with good reproductive performance and high intramuscular fat content. Youli, also a maternal line, results from crossing Landrace breed with a hybrid line made up of Chinese and European breeds, and it is a highly prolific line. Finally, the Iberian breed is a traditional 'unimproved' breed of slow growth, high fat deposition and low prolificacy but high intramuscular fat content and renowned meat quality.

A total of 16 animals × 5 tissues = 80 samples were analyzed with Affymetrix microarrays. After normalization, the resulting data were analyzed with a Bayesian linear procedure, as described in the methods section. The analysis employed allowed us to estimate the fraction of each source of variability via the variance ratios (h^2^), i.e., the variance of the effect divided by the total variance. We and others [3,5,6] have shown that mixed model methods are a powerful yet parsimonious approach for analyzing microarray data. The Bayesian approach allows us to get easily standard error estimates of all parameters in the model, including variance components and their ratios. Table 1 consistently indicates that probeset is the single most important source of variability in all cases, i.e., each gene has its own distinct expression pattern, although certainly correlated with other genes. Aside from this, the next most relevant effect is the tissue, which accounts for $h_{PT}^{2}$ ~ 10% of total variability. Neither sex nor breed exerted an overall influence on the transcriptome. These figures are concordant with a previous analysis where we studied a larger number of tissues, 16 [3]. Thus, it seems that results from Table 1 can be extrapolated to other situations, at least in the porcine species.

**Table 1:** Means (SD) of the variance ratios' posterior distributions.

**Dataseti**	$h_{P}^{2}$	$h_{PT}^{2}$	$h_{PB}^{2}$	$h_{PS}^{2}$
All	0.84 (0.08)	0.12 (0.08)	3 × 10^-3 ^(10^-4^)	4 × 10^-3 ^(10^-4^)

Males	0.86 (0.01)	0.12 (0.01)	3 × 10^-3 ^(10^-4^)	-

Females	0.86 (10^-3^)	0.11 (10^-3^)	3 × 10^-3 ^(10^-5^)	-

Large White	0.86 (10^-3^)	0.11 (10^-3^)	-	4 × 10^-3 ^(10^-4^)

Duroc	0.86 (10^-3^)	0.11 (10^-3^)	-	4 × 10^-3 ^(10^-4^)

Youli	0.86 (10^-3^)	0.11 (10^-3^)	-	4 × 10^-3 ^(10^-4^)

Iberian	0.87 (10^-3^)	0.10 (10^-3^)	-	3 × 10^-3 ^(10^-4^)

HYPO	0.98 (10^-4^)	-	3 × 10^-3 ^(10^-5^)	3 × 10^-4 ^(10^-5^)

AHYP	0.98 (10^-4^)	-	3 × 10^-3 ^(10^-5^)	3 × 10^-3 ^(10^-5^)

THYG	0.98 (10^-4^)	-	4 × 10^-3 ^(10^-5^)	10^-3 ^(10^-5^)

GONA	0.91 (10^-3^)	-	4 × 10^-3 ^(10^-5^)	0.05 (10^-3^)

GONAM	0.97 (10^-4^)	-	0.01 (10^-4^)	-

GONAF	0.95 (10^-3^)	-	7 × 10^-4 ^(10^-4^)	-

FATB	0.97 (10^-4^)	-	5 × 10^-3 ^(10^-4^)	6 × 10^-4 ^(10^-5^)

$h_{P}^{2}$: Probeset heritability; $h_{PT}^{2}$: Probeset × Tissue heritability; $h_{PB}^{2}$: Probeset × Breed heritability; $h_{PS}^{2}$: Probeset × Sex heritability.

HYPO: Hypothalamus; AHYP: adenohypophysis; THYG: thyroid gland; GONA: gonads both sexes; GONAM: male gonads; GONAF: female gonads; FATB: backfat.

When microarrays were analyzed separately by sex or breed, neither the influence of tissue nor of sex varied. That is, the ratio of *Probeset × Tissue *variance ($h_{PT}^{2}$) was constant across sexes and breeds, as was the ratio of *Probeset × Sex *variance ($h_{PS}^{2}$) across breeds (Table 1). When each tissue was analyzed separately, however, the picture changed. First, the *Probeset × Sex *variance ratio was maximum for the gonad tissue: $h_{PS}^{2}$ = 0.05 vs. $h_{PS}^{2}$ ≤ 10^-3 ^in the rest of tissues. As this ratio measures the relevance of sex in the probeset variability, this result is not completely unexpected, and agrees with previous evidence indicating the largest number of sex differentially expressed genes occurs in the reproductive organs [7]. It is far more interesting, though, the observation that the *Probeset × Breed *variance ratio was larger in male ($h_{PB}^{2}$ = 0.01) than in female gonads ($h_{PB}^{2}$ = 0.0007). That is, the breed effect was over ten times larger in the testicle than in the ovarian transcriptome. Among the tissues studied, the largest transcriptome divergence between breeds corresponded to the male gonad and the minimum, to the female gonad.

In order to test whether differential gonad development across breeds was the cause for transcriptome differences in the male gonads, we determined sex hormone levels in plasma of all animals at the time of slaughter (Table 2). There were no statistical differences neither in testosterone, the primary male hormone, nor in progesterone, a hormone produced by the corpora lutea after ovulation that can also be released from the adrenal gland after stress [8]. Male pigs secrete estradiol, primarily from Leydig cells during embryo and early days after birth [9]. Here, we found slightly elevated estradiol levels in Iberian pigs (P-value = 0.04), although they were all very low and at prepuberal levels. Thus, there was not evidence overall of large disparities in reproductive physiological stages between breeds.

**Table 2:** Sex hormone levels in serum (ng/ml) relative to Large White (LW)

Contrast	Testosterone (males)	Progesterone (females)	Progesterone (all)	Estradiol (all)
Mean	0.17 ± 0.01	1.20 ± 0.49	0.86 ± 0.34	0.016 ± 0.005

Duroc – LW	-0.01 ± 0.02	0.79 ± 0.70	0.15 ± 0.43	-0.003 ± 0.006

Youli – LW	0.02 ± 0.02	0.92 ± 0.85	0.28 ± 0.44	0.000 ± 0.006

Iberian – LW	0.01 ± 0.02	0.71 ± 0.70	0.20 ± 0.43	-0.015 ± 0.006*

* Nominal P-value 0.05

**Figure 1:**
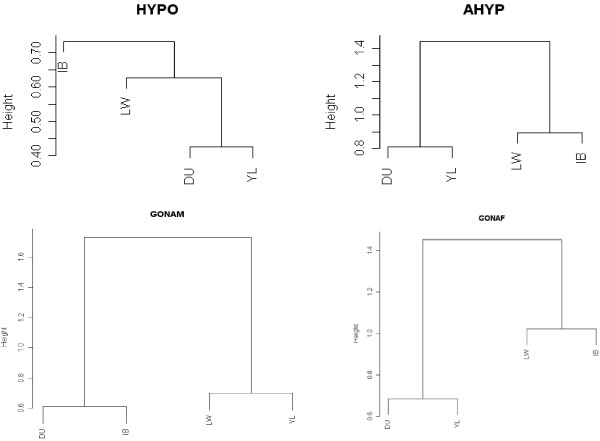
**Dendrograms between breed z-scores for a subset of tissues**. The breed z-score is a Bayesian standardized measure of expression level in that breed (see methods).

In addition to quantitative variance ratios from Table 1, we also visualized via dendrograms, that offer a convenient display of highly dimensional data. Here we used the UPGMA (Unweighted Pair Group Method with Arithmetic mean) criterion as implemented in the R-package *hclust*. Table 1 hinted that tissue is an important source of variability, and how this affects the 'relationships' between breeds can be explored with such dendrograms. Figure 1 shows the distance between breeds for a subset of tissues; the distance employed was 1-r, r being the correlation between breed z-scores across probesets (see Methods). Distances between breeds differed acording to the tissue analyzed, as can be clearly seen from comparing the scales in the vertical axes. By noting that a height of 1 corresponds to r = 0, it can be seen that correlations at hypothalamus were higher overall than for the rest of tissues. There were more differences (the trees were deeper) at male than at female gonads. Duroc and Youli were the nearest breeds for hypothalamus, adenohypophysis and ovary transcriptomes, but not for male gonads. In summary, it seems that animal breeding has targeted different tissues during the process of artificial selection, supporting a previous hypothesis [3].

**Figure 2:**
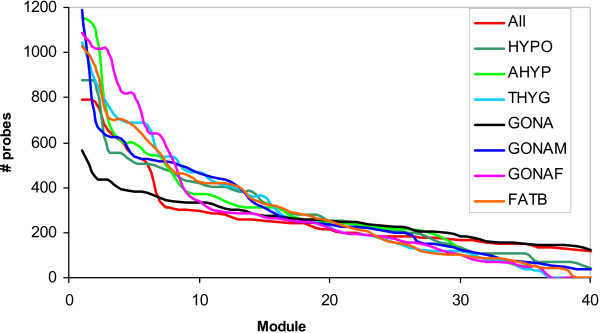
**Distribution of number of probesets per module for each tissue**.

A further aspect of interest in highly multidimensional data is connectivity, i.e., how much inter correlated are the different variable, here expression levels. To do that, we subdivided the probesets into distinct modules, where each module contains the probesets that showed maximum intercorrelation. Due to computational limitations, we ran the module detection algorithm with the 12,000 most variable probesets, approximately the number of probesets with standard deviation above the median. Figure 2 shows the number of probesets per module in the 40 largest modules. Note that the larger the size of the first modules, the larger the connectivity. Thus, the tissue rank in connectivity, measured as number of probesets included in the first five modules, was: GONAF > AHYP > THYG > FATB > GONAM > HYPO > ALL > GONA. It is interesting to remark that connectivity was minimal when gonads from both sexes are jointly analyzed and much larger when analyzed separately. This illustrates that the gonadal genetic programmes are clearly distinct in each sex.

### Differential sex expression is predominant at gonad tissues

Heritabilities in Table 1 suggest that sex is a more important source of transcriptome variability in gonads than in the rest of the tissues analyzed. But these are global measures obtained across all genes, little biological insight is provided. Sex Bayesian z-scores, in contrast, are specific to each gene; they are a quantitative measurement of how much an individual gene is differentially expressed between sexes (see Methods). Thus, the distribution of z-scores can also be used as a proxy to elucidate the level of sex bias expression, with the advantage over variance ratios that is directly interpretable in biological terms. Table 3 shows the average and the SD of sex z-scores in each tissue. Statistics are reported for all probesets and for the 100 most extreme probesets. The tissue with largest sex bias overall was the gonad, as expected, and it was also the most variable. Sex bias was much more marked for gonads than for the rest of tissues when only the 100 most extreme probesets are used. These results correlate well with estimates of the *Probeset × Sex *heritabilities (${\left({\sum_{j=1,4}\left[z_{ij}-{\bar{z}}_{i}\right]}^{2}/3\right)}^{1/2}$, Table 1). At a false discovery rate FDR = 0.05, the number of significant sex – biased probesets was 1714 (all tissues), 0 (HYPO), 250 (AHYP), 20 (THYG), 5154 (GONA) and 0 (FATB). In summary, the most sex biased tissues were the gonads, followed by the adenohypophysis and the thyroid gland. The least sex-biased tissues were hypothalamus and fat.

**Table 3:** Means (SD) of sex Bayesian z-scores, absolute values, for each tissue.

**Probesets**	**All**	**HYPO**	**AHYP**	**THYG**	**GONA**	**FATB**
All	1.12 (1.91)	0.18 (0.18)	0.57 (0.90)	0.37 (0.47)	1.93 (2.82)	0.20 (0.26)

Largest 100*	17.1 (15.0)	1.06 (0.10)	8.80 (3.75)	3.82 (1.70)	22.9 (4.14)	1.94 (0.41)

* In each tissue.

HYPO: Hypothalamus; AHYP: adenohypophysis; THYG: thyroid gland; GONA: gonads both sexes; GONAM: male gonads; GONAF: female gonads; FATB: backfat.

**Figure 3:**
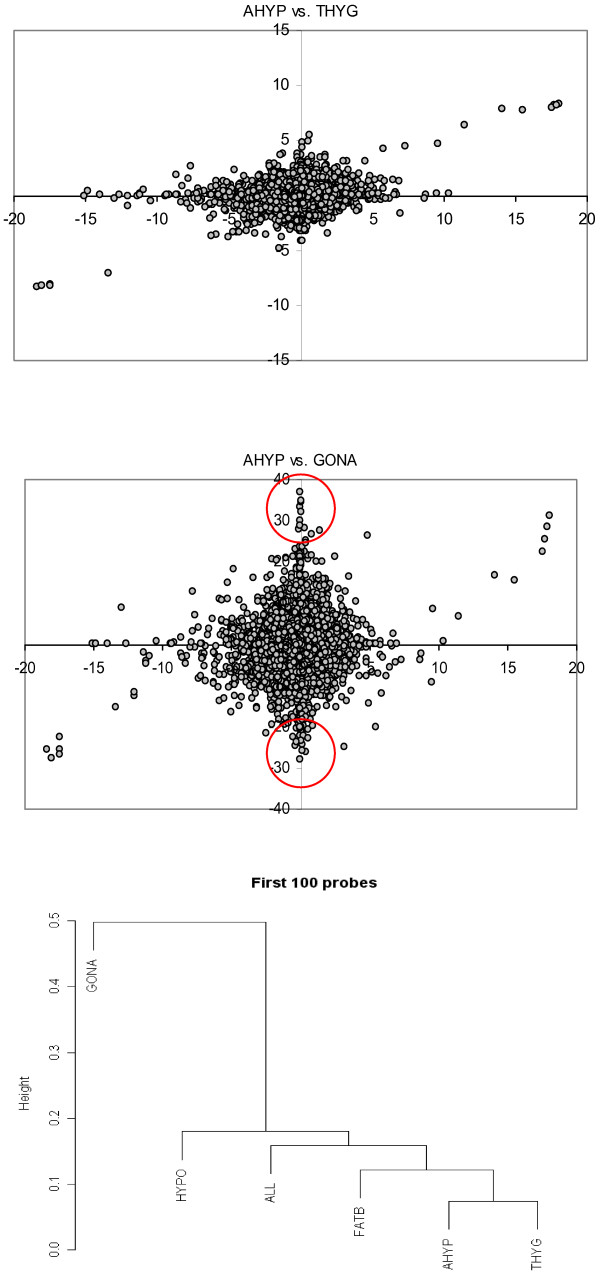
**Sex z-scores across tissues**. The sex z-score is a Bayesian standardized measure of expression difference between sexes (see methods). Each dot in top and middle figures corresponds to a different probeset. Top: Adenohypophysis (abscissa) vs. thyroid gland (ordinate) sex z-scores. Middle: Adenohypophysis (abscissa) vs. gonad (ordinate) sex z-scores; the encircled dots correspond to highly biased probesets in gonads that show no bias in adenohypophysis. Bottom: Dendrogram of the sex z-scores in different tissues corresponding to the 100 most sex biased probesets. The vertical scale is the distance between points, i.e., one minus the Pearson correlation.

How likely is that sex – bias is conserved across tissues, i.e., that a sex – biased gene in a given tissue is also sex – biased in another tissue? A general response to this question can be approximated by the correlation of sex z-scores between tissues. Table 4 (upper diagonal) shows that this correlation was rather small when all probesets were considered, that is, there was no common pattern of sex – bias expression. The only exception was between z-scores at the gonad and that obtained when all tissues were jointly analyzed (r = 0.83). But this occured because the z-score across all tissues was heavily influenced by the pattern in gonads, simply because it was in this tissue where the largest sex bias was found (Table 3). It is more meaningful to consider a subset of genes, those with largest sex – bias across all tissues (lower triangle in Table 4). A completely different picture emerges now. Overall, correlations were very high between tissues, meaning that a highly sex – biased gene in a given tissue tends also to be sex – biased in other tissues. Yet, it was intriguing that correlations between the gonad and the rest of tissues were much lower now, hinting that highly sex – biased genes in the gonads were at least partially different from those in the rest of tissues. This heterogeneous gonad sex – programme was visible by plotting the z-scores. For instance, the correlation between AHYP and THYG z-scores was 0.93 for the extreme sex – biased probesets and 0.24 across all probesets (Table 4). The z-scores were plotted in Figure 3 (Top). Note that, save for a few probesets, the relationship between extreme z-scores was linear and in agreement with the high correlation found, whereas it was not when all probesets were considered. As for the gonads, Figure 3 (Middle) plots the AHYP vs. GONA z-scores. In this case, there was a considerable number of probesets with extreme bias in the gonads but not in the adenohypophysis, those encircled in red. As a result the correlation between z-scores of the most sex – biased probesets is low. An UPGMA cluster did show also that the gonad genetic programme, in terms of sex bias expression, was clearly distinct from the rest of tissues analyzed (Figure 3 Bottom).

**Table 4:** Correlation of sex Bayesian z-scores between tissues

	**All**	**HYPO**	**AHYP**	**THYG**	**GONA**	**FATB**
All	-	0.24	0.43	0.38	0.83	0.33

HYPO	0.79	-	0.16	0.19	0.03	0.12

AHYP	0.86	0.85	-	0.24	0.09	0.15

THYG	0.85	0.84	0.93	-	0.06	0.20

GONA	0.77	0.47	0.42	0.42	-	0.07

FATB	0.81	0.80	0.88	0.88	0.43	-

Upper diagonal, correlation across all probesets; lower diagonal, correlation across the 100 most differentially expressed genes in all tissues. HYPO: Hypothalamus; AHYP: adenohypophysis; THYG: thyroid gland; GONA: gonads both sexes; GONAM: male gonads; GONAF: female gonads; FATB: backfat.

This distinct genetic programme in the gonads, one would expect, should consist of genes primarily involved in gametogenesis. To investigate this further, we selected the 100 most biased probesets in the gonads and, among those, we ranked the most biased probesets in AHYP and THYG. The complete list is in Additional File 2, but nine probesets stood out as highly sex – biased across all three tissues, whereas the 91 remaining probesets did not exhibit any pronounced sex bias expression neither in AHYP nor in THYG. The nine probesets corresponded to genes *DDX3Y, EIFS23, FAM5C, EIFIAY, DENND4A, PTPRM, LPHN2, CLOCK *and *TMSB4X*. These nine genes were among the 10 most differentially sex expressed genes that we obtained in a previous work [3], where 16 tissues in four animals were analyzed. Some of the genes were confirmed by quantitative real time PCR (QRT-PCR). They are also among the most sex biased porcine genes identified in independent studies [10]. We also performed a gene ontology analysis of the remaining 91 probesets which did show, as presumed, that spermatogenesis genes were over represented (P-value = 0.03) but also were other processes: multicellular organism development (P = 0.03), hemophilic cell adhesion (P = 0.01) or synaptic transmission (P = 0.04). Thus, although gametogenesis partially explains the distinct sex-bias programmes between gonads and the rest of tissues, it does not explain the full story. There is not a simplistic explanation or a general common role for specific gonad sex – biased genes.

We also carried out a differential gene ontology (GO) study with the most sex biased probesets in the gonads, irrespective of whether they were also sex – biased in other tissues. Initially, we aimed at studying the 5154 sex biased probesets detected at FDR = 0.05 but this was not feasible computationally. Thus, we selected the top 1700 genes, the number of selected genes when all tissues are analyzed. The most significant and numerous GO classes are in Additional File 3. Overall, most GO categories were related to development, which makes sense because ovaries and testicles follow different development trajectories since early embryogenesis, from about four weeks of embryo age in pigs [11]. As expected, there was also an overrepresentation of spermatogenesis and male gonad development, but also lactation. An interesting observation was the excess of genes related to the MAPKKK cascade (*MAP3K5, MAPK1, FGFR3, RAPGEF2, NF1, AGT*), one of the most important signalling pathways in the cell. All of these genes are also involved in development, in particular, *AGT *(angiotensinogen) plays a role in female pregnancy and in ovarian follicle development [12,13].

### Breed differences

The study of breed differences at the transcriptome level is fundamental to elucidate the impact of selection on gene expression, and thus on gene regulation. As mentioned, the ratio $h_{PB}^{2}$ or *Probeset × Breed *heritability is a summary of the breed influence on the transcriptome. Although the overall breed influence was much smaller than that of tissue or the probeset itself (Table 1), it was a remarkable observation that $h_{PB}^{2}$ was maximum in male gonads and minimum at the female gonads: 0.01 vs. 0.0007, i.e., at least 10 times larger in testicles than in ovaries. Note that the SD of these figures are very small (= 10^-4^) and thus the two estimates are clearly distinct. To gain further insight and to assess the influence of each individual probeset, we computed the standard deviation of the breed Bayesian z-scores, i.e., for probeset i SD_zbreed, i _= $h_{P}^{2}\;=\;\sigma_{P}^{2}/\;\sigma_{Y\;}^{2}$, where *z*_*ij *_is the breed z-score of probeset i at breed j. This is a rough measure of expression variability across breeds but allows us to carry out a ranking among probesets in terms of how much do their expression differ between breeds. Additional File 4 shows the mean SD_zbreed _for each tissue, the complete list of breed z-scores is in Additional File 5. The z-score breed variability was larger in the male gonads than in the rest of tissues, in agreement with the variance component analysis from Table 1. Only when all tissues or both gonads were considered jointly was variability larger than in male gonads. The relation between breed and sex bias expression is discussed in the next section.

Considering that a large heterogeneity in SD_zbreed _was observed between tissues, we investigated whether there were any genes that consistently showed large breed variability across several tissues. The rationale was that these genes could be primary targets of artificial selection or provide clues about main metabolic routes responsible for breed differences. To study this, we selected the probesets that were among the 200 top most variable probesets (i.e., highest SD_zbreed_) in at least four tissues. This criterion was fulfilled by a total of 19 probesets (Table 5). The majority of corresponding genes was involved in several development and cell cycle processes, including apoptosis. Six of the genes in Table 5 were among the 31 most distinct genes between Large White and Iberian pigs that we found in a previous study [3], these genes are marked with a star (*) in Table 5. This is a highly significant (P << 10^-6^) and remarkable overlap considering that i) the number of breeds was doubled here in relation to [3], ii) the number of tissues was quite different between both studies, 16 in [3], five here; gonads, the most variable tissue, was not studied in [3], and iii) the statistical inference method was different.

**Table 5:** Genes that are among the 200 most variable in at least four tissues

**Name**	Symbol *	**GO Biological process**	**z_LW_**	**z_DU_**	**z_YL_**	**z_IB_**	**z_SEX_**
Lysosomal trafficking regulator	*LYST*	Cellular defense	9.1	3.3	8.0	-20.7	8.4

phosphatidylethanolamine N-methyltransferase	*PEMT*	Cell proliferation	18.4	-12.6	0.1	-6.2	2.0

Small VCP/p97-interacting protein	Q8NHG7*	-	-10.5	-8.7	0.7	18.7	0.7

forkhead box F1	*FOXF1*	Organ morphogenesis, lung and gut development	5.2	-7.3	12.1	-9.9	1.3

Death inducer-obliterator 1	DATF1*	Apoptosis	12.6	-4.3	-3.8	-4.6	0.4

Major histocompatibility complex class B	HLA-B*	Antigen processing	-4.2	-4.6	-2.4	11.1	4.0

Heterogeneous nuclear ribonucleoprotein H2	*HNRPH2*	RNA binding	3.0	2.0	-11.7	7.2	4.8

Vesicle trafficking protein homolog B	*SEC22L1*	ER to Golgi transport	-1.6	6.7	-9.7	4.3	2.0

Microtubule-associated protein 6	*MAP6*	Negative regulation of microtubule depolymerization	4.7	-6.5	11.3	-9.3	8.9

Rho-associated, coiled-coil containing kinase 1	*ROCK1*	Actin cytoskeleton organization and biogenesis	-4.9	-4.7	14.4	-4.67	-5.7

Family with sequence similarity 92, member A1	*FAM92A1*	-	-10.2	14.3	0.8	-4.7	3.3

Frizzled homolog 4 (Drosophila)	*FZD4*	Multicellular organism develop, wnt signaling pathway	-14.2	12.1	5.6	-3.6	2.0

Opticin	*OPTC*	Protein binding	2.0	-0.9	3.0	-4.0	3.1

Armadillo repeat containing, X-linked 1	ARMCX1*	Development, maintenance of tissue integrity	-13.6	6.5	0.5	6.5	3.6

Lipopolysaccharide-induced TNF factor	*LITAF*	Apoptosis	-3.9	7.4	-7.9	4.5	7.7

Vasoactive intestinal peptide receptor 2	*VIPR2*	Cell cell signaling/G-protein coupled receptor protein signaling	4.1	4.7	-15.5	6.8	1.9

Amyloid beta (A4) precursor-like protein 2	*APLP2*	G-protein coupled receptor protein signaling	-4.5	-3.9	14.4	-5.8	2.3

Tousled-like kinase 2	TLK2*	Cell cycle/chromatin assembly	12.6	-4.3	-3.5	-4.9	2.9

Immunoglobulin heavy constant mu	IGHM*	Antigen binding	-1.2	2.2	-0.9	0.1	4.3

* Also found differentially expressed between breeds in Ferraz et al. [3]

The most extreme breed for each of the geneprobes is shaded in Table 5. Although the Youli synthetic breed seems 'enriched' in extreme probes, may be because of its highly heterogenous backgroung, it is of interest to study the extreme probes between Iberian (a non selected breed) vs. the rest of breeds, which have undergone a rather intense artificial selection process. We did that for all tissues jointly and for each of individual tissues except the gonads. In all cases, the gene LYST, involved in cellular defense, was the most extreme Iberian probe. We also looked for enriched ontology categories among the most extreme probes. When all tissues are examined together, the most 100 extreme genes were enriched in defense (antigen processing and response) and development (endodermal cell fate commitment, brain morphogenesis, mast cell biogenesis). As for each tissue independently, thyoroid gland was enriched in defense genes, whereas back fat or hypothalamus showed, additionally, an excess of muscle development genes.

A potential limitation of considering each gene individually, e.g., Table 5, is that there might not be enough power in the data to detect all influential genes. Taking into account the high inter correlation between expression levels should help to improve upon this. For this purpose, we studied whether there was a connection between modularity, i.e., gene coexpression, and bias, i.e., differential expression across breeds. We reasoned that, assuming that genes in a given module tend to work coordinately, because their expression is highly correlated, an excess of breed – biased genes in a module should help to uncover metabolic pathways that have been primarily affected by breed divergence. As a result, these biological processes may be more easily influenced by selection, either artificial or natural selection. Specifically, we studied whether there was an excess of the top 100 most variable genes in any of the modules detected (Figure 2). Four modules were particularly 'enriched' in breed biased genes (P << 10^-6^). These were modules 4, 14 and 29 in male gonads and module 25 in back fat (Table 6). Then, we studied whether any specific GO biological process was overrepresented in *all *genes for that module, i.e., not only those that showed high breed variability. Table 6 shows that each module was enriched in different GO processes. The largest module (number 4 in GONAM) was particularly enriched in spermatogenesis whereas the rest of the modules were 'specialized' in other processes, muscle functioning or development (modules 14 GONAM and 25 FATB) or several metabolic processes.

**Table 6:** Over represented Gene Ontology (GO) categories in modules with largest discrepancy between observed and expected number of probesets

**Tissue**	**Module**	**n_OBS_**	**n_EXP_**	**GO Name**	**P-value**	**FDR**
Male Gonad	4	47	5	Spermatogenesis	10^-5^	0.05

				Spermatid development	0.002	0.37

				Spindle organization	0.002	0.07

	14	13	2	Muscle cell differentiation	10^-5^	0.07

				Mitotic sister chromatid cohesion	10^-4^	0.32

				Negative regulation of transcription	0.003	0.77

	29	12	2	Aldehide metabolic process	0.003	0.34

				RNA metabolic process	0.006	0.34

				Glycogen metabolic process	0.007	0.34

Backfat	25	34	1	Striated muscle contraction	< 10^-5^	10^-4^

				Regulation of striated m. contraction	10^-4^	0.03

				Ventricular cardiac muscle morphogenesis	0.001	0.30

Module: The number of probesets per module is shown in Figure 2

n_OBS_: Number of probesets, among those with 100 highest SD_zbreed_, observed in the module

n_EXP_: Number of probesets expected in the module

FDR: false discovery rate

### Is there a link between breed and sex biased expressions?

**Figure 4:**
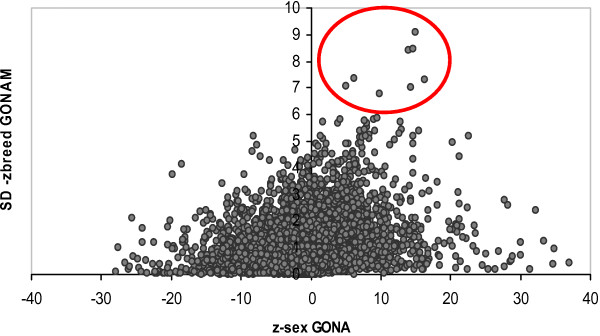
**Sex bias vs. variability of breed z-scores in male gonads**. In abscissas, the sex z-score in gonads; in ordinates, the standard deviation of breed z-scores in the male gonads, i.e., a probeset with a high ordinate value is a probeset whose expression level in testicles varied largely between breeds. Each dot corresponds to a different probeset. The probesets encircled are those with largest variability across breeds which, as can be seen, are male biased but are not the most sex biased.

The fact that gonads were the most sex biased tissue, and that the most variable genes between breeds were expressed in the male gonads, leads to the obvious question of whether the same genes are involved in both sex and breed biased expression. However, the relationship was not very strong. The correlation between |z-sex_GONA_| and SD_zbreed, GONAM _across probesets was 0.38. This value is rather low, it corresponds to a coefficient of determination r^2 ^= 0.14. Figure 4 shows that there was not a constant relation between breed variability and differential sex expression in the gonads. Note, nevertheless, that the most variable genes (highest SD_zbreed_, encircled) were male sex biased, although they were not the *most *sex biased genes. Table 7 lists the 15 most breed variable genes in the testicles, together with their sex z-score. At least four genes, almost 25%, were directly involved in spermatogenesis. At least one gene (*MAP3K5*) was directly involved in the MAPKKK cascade, one of the overrepresented GO categories among sex biased genes (Additional File 3). The most variable gene and that with highest sex bias was *PHKA*, a key regulatory enzyme of the glycogen metabolism. Note that, despite being located on chromosome X, this gene shows a marked male biased expression. Interestingly, it is known that chromosome X harbors an excess of sex biased genes [14].

**Table 7:** The 15 most variable genes among breeds in the male gonads together with their sex z-score.

**Name**	**Gene/Probeset**	**GO biological process**	**SD_zbreed, GONAM_**	**z_sex, GONA_**
Phosphorylase kinase, alpha 1	*PHKA1*	Muscle glycogenosis (X located)	9.1	15.0

Cysteine-rich secretory protein 1	*CRISP1*	Spermatogenesis	8.5	14.7

Mitogen-activated protein kinase kinase kinase 5	*MAP3K5*	MAPKKK cascade, apoptosis	8.4	14.0

Lysosomal associated protein transmembrane 4 beta	*LAPTM4B*	Transport	7.3	6.3

Caspase 8, apoptosis-related cysteine peptidase	*CASP8*	Apoptosis	7.3	16.3

Y box binding protein 2	*YBX2*	Spermatogenesis	7.1	5.1

coiled-coil domain containing 71	*CCDC71*	-	7.0	14.2

Protamine 1 (testis specific)	*PRM1*	Spermatogenesis	6.8	9.9

Unknown	Ssc.18340.1.A1_at	-	5.9	9.4

Progestin and adipoQ receptor family member VII	*PAQR7*	Steriod binding	5.8	9.0

Aldehyde dehydrogenase 1 family, member A1	*ALDH1A1*	Aldehide metabolic process	5.8	4.2

Protein phosphatase 1E	*PPM1E*	Protein amino acid dephosphorylation	5.8	7.7

Sperm autoantigenic protein 17	*SPA17*	Spermatogenesis, fertilization	5.7	1.7

Aquaporin 8	*AQP8*	Water transport	5.7	12.8

Unknown	Ssc.11206.1.A1_at	-	5.7	3.9

SD_zbreed, GONAM _is the standard deviation of breed z-scores in the male gonads, where the breed z-score is a standardized measure of the expression level in each breed; z_sex, GONA _is the sex z-score, a standardized measure of expression difference between sexes. A positive value indicates a male overexpression with respect to females.

As for the most variable genes across several tissues (Table 5), there was an excess of male biased genes, except *ROCK1*, all were overexpressed in males. Again, they were not the most sex biased genes. Note that none of the 15 most variable genes among breeds in the male gonads (Table 7) were also present in Table 5, that is, the most variable genes among breeds in the gonads were not the most variable across all tissues. To summarize, the most variable genes among breeds were predominantly male biased. However, the most variable genes were not the most sex biased. An important percentage of the most variable genes were involved in spermatogenesis (Table 7), suggesting that artificial selection targets this biological process, either directly or indirectly.

## Discussion and Conclusion

Animal breeding has resulted in breeds that are extremely diverse for a large number of traits. These phenotypic differences are influenced by DNA variants and mediated by distinct transcriptome programmes across breeds. The dissection of the transcriptome breed differences will thus largely illuminate the physiological and genetic causes underlying artificial selection and breed differentiation. Here we reasoned that many changes observed in target selection tissues, i.e., muscle and fat, might actually be due to signals external to the tissue itself, notably through the endocrine system. In addition, and despite the relevance of the endocrine system in animals, the knowledge of its transcriptome is rather scarce.

Among the many statistical approaches employed to analyze microarrays e.g. [15], here we have adopted a Bayesian approach that is closely related to mixed model methods [16]. The main advantages of these approaches are their modeling flexibility while allowing the whole dataset to be analyzed simultaneously. Both characteristics are important to contrast a number of biological hypotheses with a minimum standard error and, consequently, maximum power. In addition the Bayesian method provides an exact measure of error for variances and variance ratios, whereas convolute approximations are needed in the classical mixed model method. There are also potential hindrances in the analyses reported here. The main one is that variance homogeneity is assumed, unaccounting for differences in variability across probes other than the effects included in the model. For instance, a gene whose expression level is very low across all tissues is less variable than a gene expressed in some tissues and switched off in another tissues. There is a rich literature on heteroskedasticity, especially within the Bayesian paradigm. However, accounting for variance heterogeneity obliges to fit a distinct variance for a gene or a group of genes, which can be extremely hard to compute given the large number of genes in microarrays. An alternative is to analyze each gene separately, but this is also undesirable because many parameters are estimated and the risk of false positive increases.

Nevertheless, the models used here fitted the data quite well, as evidenced by the high heritabilities reported (Table 1), an there are several relevant conclusions that can be drawn from our study. First, we show that probeset is by far the most influential factor, accounting for at least 85% of total variability, whereas tissue explains in the order of 10%. Breed and sex contribute only marginally to total variance in the transcriptome. There are no differences across breeds nor between sexes in this respect. These results agree extremely well with a previous study from our group, although there we employed a different statistical methodology and we analyzed 16 tissues in a smaller number of animals (four) [3]. Although sex and breed were, globally, much less relevant than the probeset effect, sex and breed do influence largely the expression of a subset of genes: those with most extreme z-scores.

Also importantly, we report a strong link between sex – bias and breed variability, which is not caused by large differences in reproductive development (Table 2). The male gonad is the tissue with largest breed heritability ($h_{PB}^{2}$, Table 1). This result is coherent with several independent observations. First, many of the genes that have been identified as undergoing selection in the human and other species are involved in spermatogenesis [17-19]. It is plausible then that artificial selection and breed divergence, which operates through the same mechanisms as evolution, affects also spermatogenesis. Second, modern breeding in livestock targets primarily the male because a sire can leave much more offspring than a dam, and thus selection intensity is usually much higher in males than in females. This would help to explain why sex biased and breed biased genes partially overlap (Table 7, Figure 4). And third, recent work in Drosophila [20] and references therein have confirmed that sex biased genes exhibit a faster rate of evolution than non biased genes; in addition, male biased genes show a stronger signal of adaptive selection than female biased genes. Nevertheless, although the most breed – biased genes tend to be also sex biased, the most sex biased genes are not among the most breed biased genes. Thus, these two phenomena are inextricably but only partially linked. Similarly, not all genes among those with largest breed – variability are involved in spermatogenesis (Table 7, Additional File 5). Thus, the high breed heritability in the male gonads cannot be explained solely by changes in spermatogenesis. This is certainly an area meriting further research.

A worth noting observation is an elevated number of myogenesis related processes among genes involved in breed differentiation (Table 7). The muscle – the major component of the meat – is the tissue that has been the main target of artificial selection in the pig. We have previously shown [3] that a number of genes involved in myogenesis were differentially expressed in both tissues. Thus, the excess of muscle development genes in fat and gonads might simply reflect a pleiotropic change caused by a primary effect in muscle. Thus, our initial hypothesis that breed transcriptome differences might affect primarily the endocrine system should be reevaluated, as is not fully supported by our experimental results. In fact, it is quite remarkable that both sex and breed differences at the hypothalamus, one of the key endocrine organs, is smaller than in the rest of tissues studied (Table 3, Additional File 4). Certainly, the endocrine system plays a fundamental role in animal's physiology and consequently in breed and sex differences, but may be transcriptome differences are more pronounced at development stages other than that studied here or affect a very small subset of genes.

As expected in the light of previous research in the pig and in other species, e.g., [3,7,21], we find extensive evidence of sex biased probesets. Not surprisingly, the gonads are the most sex biased tissue overall (Table 3, all data are in additional file 2). Globally, the most sex biased genes are also sex biased across a range of tissues, except in the gonads (Table 4, low triangular, and Figure 3). Most sex biased genes in Table 4 were identified previously by us and by an independent group, and some were confirmed by quantitative real time PCR [3,10]. Several of the gonad sex biased genes identified here are known to be involved in gonadal development in mice and pigs [22,23], like *LHX9*, *PODL*, GATA4, *AMH *(z_sex _= 6.7 in gonads, z_sex _~ 0 in the rest of tissues), *SOX9 *(z_sex _= 12.0 in gonads, ~0 in the rest of tissues). In contrast, we do not find any sex bias for sex determining region (*SRY*, z_sex _= 0.08), which initiates the sex differentiation cascade, probably because its temporal expression is very narrow, 10 – 12 days post coitum in the mouse [22]. Follistatin, a glycoprotein forming part of the inhibin-activin-follistatin axis that plays an important role in follicular development within the ovary, is highly overexpressed in ovary (z_sex _= -16.7) but no significant bias appears in the rest of tissues analyzed. The most female biased gene, nonetheless, is protein tyrosine phosphatase receptor type M (*PTPRM*), an important signaling molecule that regulates cell growth and differentiation. This gene was already identified in a previous study [3] as being also strongly female biased.

As genes work coordinately and thus their expression levels are correlated, considering gene modules should be a more powerful approach than analyzing each gene separately. We observe that connectivity varies across tissues (Figure 2) and that the least connected transcriptome occurs when gonads of both sexes are jointly analyzed. This is likely a result of large heterogeneity in expression patterns between ovaries and testicles even before puberty. But, for our purposes, the main use of detecting modules was combining expression bias and connectivity in order to increase power and discover more subtle signals that may not be evident when studying each gene in isolation [24]. Several approaches can be envisaged to attain this. Here, first we identified sets of highly correlated genes (modules) within each tissue using standard techniques [25], followed by an assessment of whether any module was enriched in breed biased genes. Finally, we looked for over represented gene ontologies among all genes in that module. We found that different modules were enriched in specific ontologies (Table 7), reflecting the modularity of gene expression. Importantly, we identify a series of biological processes (spermatogenesis, muscle differentiation and several metabolic processes) that have been the likely target, direct or indirect, of artificial selection. The next logical step will be to verify whether genes that have been the target of selection (showing evidence, e.g., of a selective sweep) in the pig are enriched in these gene ontologies. At least in humans, there a significant excess of genes undergoing natural selection are involved in spermatogenesis [26].

## Methods

### Animal material

Sixteen animals, four from each of four breeds, Large White (LW), Duroc (DU), Youli (YL) and Iberian (IB) piglets were sampled. These breeds represent a wide genetic variability in current pig breeding schemes. There were two males and two females per breed except in Youli, represented by three males and one female. Animals were bought from three breeding companies and transferred to the University experimental farms at weaning, i.e., aged one month approximately. Pigs were housed simultaneously, fed the same diets during the fattening period, that lasted two months, and were weighed at weekly intervals. At the time of slaughter, the average ages were 87, 83, 80 and 89 days for Large White, Duroc, Youli and Iberian pigs, respectively. Their mean live weights at that time were 27.2 (LW), 23.1 (DU), 18.9 (YL) and 17.4 kg (IB).

Animals were euthanized, after 24 h fasting, by an overdose of intravenous sodium thiobarbital. At necropsy, tissue samples were collected, snap frozen in liquid nitrogen and stored at -80°C. The average time gap between euthanasia and tissue collection was ~15 minutes, maximum time was 25 minutes. The tissues collected were hypothalamus (HYPO), adenohypophysis (AHYP), which was separated from the neurohypophysis, thyroid gland (THYG), gonads (GONA) from both sexes, males (GONAM) and females (GONAF), and back fat tissue (FATB). The hypothalamus included the mamillary body and grey tubercle but excluded the chiasma opticum. Throughout this work, each sample was identified by the acronym of the tissue followed by the animal id, e.g., FATB_LWF1 refers to back fat tissue from female 1 Large White. All procedures were approved by the Ethical and Animal Welfare Committee of the *Universitat Autònoma de Barcelona*, in accordance with the guidelines of the Good Experimental Practices.

### RNA extraction and microarray hybridization

Total RNA was extracted from 100 mg tissue using the RiboPure™ kit (Ambion, Austin, USA) according to the manufacturer's protocol. RNA was quantified with the NanoDrop ND-1000 spectrophotometer (NanoDrop Technologies, Wilmington, USA) and the RNA integrity was assessed by Agilent Bioanalyser 2100 and RNA Nano 6000 Labchip kit (Agilent Technologies, Palo Alto, USA). Due to high variation in concentrations of the total RNA obtained in different tissues, all samples were concentrated and cleaned using the RNAeasy MiniElute Cleanup kit (Qiagen, Basel, Switzerland) obtaining final concentrations between 500 and 1000 ng/μl.

A total of 80 microarrays (16 animals × 5 tissues) were hybridized and scanned at the *Institut de Recerca Hospital Universitari Vall d'Hebron *(Barcelona, Spain). Briefly, the cDNA synthesis was undertaken with 5 μg of total RNA, labelled with biotin and hybridized to individual high-density oligonucleotide microarray chips (GeneChip^® ^Porcine) from Affymetrix (Santa Clara CA) containing a total of 23,935 probeset sets, representing 20,201 *Sus scrofa *genes, 11,265 of these genes were annotated by Tsai et al. (2006). The hybridization was done according to Affymetrix standard protocols and microarray expression data were generated with GeneChip Operating Software (GCOS). As the annotation provided by the manufacturer is not too detailed, the results in this work are based in the annotation developed by [27]. The complete data set, both GCRMA and original CEL files, are available at Gene Expression Omnibus (GEO) under accession number GSE14739. The original material, tissue conserved at -80 C, is also available on request. Contact the authors for details.

### Data processing and statistical analysis

Quality control of CEL files was done with the *Affy *package of bioconductor [28]: RNA degradation and the raw data distribution were ascertained. All CEL files were normalized simultaneously with the GCRMA procedure [29]. After normalization, a Bayesian approach was adopted for statistical inference. The full model employed was:

(1) *y*_*ijkgl *_= *Tissue*_*i *_+ *Breed*_*j *_+ *Sex*_*k *_+ *Probeset*_*g *_+ *PT*_*gi*_*+ PB*_*gj *_+ *PS*_*gk *_*+ e*_*ijkgl*_,

where *PT*, *PB *and *PS *stand for the probeset × tissue, probeset × breed and probeset × sex interactions, respectively. The subscript *l *refers to l-th individual. In the Bayesian paradigm, all effects are random. Here we used uninformative flat priors for all parameters. We defined the following heritabilities or ratios of variances: $h_{PT}^{2}\;=\;\sigma_{PT\;}^{2}/\;\sigma_{Y\;}^{2}$, $h_{PB}^{2}\;=\;\sigma_{PB\;}^{2}/\;\sigma_{Y\;}^{2}$, $h_{PS}^{2}\;=\;\sigma_{PS\;}^{2}/\;\sigma_{Y\;}^{2}$ and $\sigma_{Y\;}^{2}=\;\sigma_{P\;}^{2}+\;\sigma_{PT\;}^{2}+\sigma_{PB\;}^{2}+\sigma_{PS\;}^{2}+\sigma_{\text{e}}^{2}$, where the denominator is the total phenotypic variance, i.e., ${\left({\sum_{j=1,4}\left[z_{gj}-{\bar{z}}_{g}\right]}^{2}/3\right)}^{1/2}$. We also analyzed data subsets, e.g., only females or males, or each breed or each tissue separately. In these instances, appropriate effects were deleted from model (1), e.g., *Sex *and *PS *are removed when data from a single sex are analyzed. Bayesian analyses were carried out using standard theory [30]. A Gibbs sampling approach was performed with a home made program (A. Legarra, INRA, France, personal communication). Priors were flat bounded between two very large numbers (-10^6 ^– 10^6^); given the large amount of data, priors should have a minor influence here. We employed 10,000 iterates with 2000 burning initial iterates and discarding every 50 to minimize autocorrelation. Note that the dimension of the system in (1) is huge, it used 23,935 probesets × 80 samples ~ 1.9 10^6 ^records and contained 287,229 equations. Total CPU time was in the order of three days on a Linux Itanium server. Most of time was spent reading the data and building the equations via linked lists rather than in the Gibbs sampling process itself.

Bayesian methodology offers several advantages over more traditional least square or maximum likelihood approaches and these will not be discussed here [30,31]. It suffices to mention that the output of the Gibbs sampler are values that follow the marginal posterior distribution of each of the t parameters in the model, p(θ_t_|y), and thus it is straighforward to compute any desired statistics from that distribution taking into account uncertainty on the rest of parameters in the model. Here we report the mean and SD of the posterior distributions of the random components ratios (*h*^2^). We also defined the Bayesian z-score that, in analogy to the usual z-score, was obtained from E(θ_t_|y)/SD(θ_t_|y). We computed sex and breed z-scores; the sex z-score for g-th probeset is E [(*PS*_g1 _- *PS*_g2_)|y]/SD [(*PS*_g1 _- *PS*_g2_)|y] where subscripts 1 and 2 refer to males and females, respecively. Similarly, the j-th breed z-score for g-th probeset is defined as *z*_*gj *_= E(*PB*_gj_|y)/SD(*PB*_gj_|y). A rough measure of how much the expression varies across breeds is computed as the standard deviation of the breed Bayesian z-scores, i.e., for probeset g SD_zbreed, g _= $h_{P}^{2}$. We also computed an analogous False Discovery Rate statistics [32]. We obtained this from the sex Bayesian z-scores; given that these z-scores follow a N(z-score,1) distribution, we can compute the probability that z-score > 0 and assimilate these to P-values. The complete list of z-scores is available at additonal files 4 and 5. Thoroughout, we employed dendrograms to visualize multivariate results using the *hclust *R function. The distance chosen was one minus the Pearson correlation (r) across variables, this distance is bound between 0 (r = 1) and 2 (r = -1).

As genes function in concerted action, which is best described by networks, we studied differential connectivity and checked whether most differentially expressed genes belonged to a specific group of highly co-regulated genes (a module). To do that, we characterized the number of modules using the approach in [25] employing distance 1-r and a cut level of 95%; the minimum number of probesets per module was set to 30. Due to computing constraints, we analyzed only the 12,000 probesets with higher SD than the median. We identified modules using all data jointly or for each tissue separately. For gonads, we did the analysis with both sexes pooled and within sex. In order to identify a potential connection between modularity and bias (differential expression), we studied whether any of the modules contained a larger number of probesets than expected. Suppose a list of n_d _probesets are either breed or sex biased, and, among them, a subset of n_OBS _probesets belong to module j. In addition, suppose that n_j _is the total number of probesets in the module j. The expected number of probesets in the module is simply n_EXP _= n_d _× n_j_/12,000 (because we have selected 12,000 probesets). This value is corrected when not all n_d _probesets are among the 12,000 employed for the module analysis. We computed the chi-squared statistics, (n_OBS _- n_EXP_)^2^/n_EXP_, and the associated P-values. We did this for each module and tissue separately.

Gene ontologies and GO over representation were analyzed with onto-tools [33].

### Hormonal measurements

Hormonal concentrations were measured in duplicate in plasma using commercial testosterone (EIA #402510, Neogen Corporation, Lexington, USA), estradiol (EIA #402210, Neogen Corporation) and progesterone EIA kits (EIA #402310, Neogen Corporation). All assays were conducted following the manufacturer's protocol. The assays were validated for pig plasma by demonstrating that serial dilutions of plasma were parallel to the displacement curve for the reference standards. Hormone standards spiked with pig plasma produced accurate results of hormone recovery (102.0 ± 3.6% to 106.5 ± 9.2% in 12 assays, *r*^2 ^= 0.97).

## Availability

The data used in this study have been deposited in GEO under accession number GSE14739. The original material (tissue frozen at -80 C) is also available on request. Contact the author for details.

## Abbreviations

DU: Duroc pig breed; FDR: False Discovery Rate; GO: Gene Ontology; HPT: hypothalamic-pituitary-thyroid axis; HPTA: hypothalamic-pituitary-gonadal axis; IB: Iberian pig breed; GCRMA: GC Robust Multiarray Average; LW: Large White pig breed; QRT-PCR: Real Time Quantitative Reverse Transcription Polymerase Chain reaction; SD: standard deviation; UPGMA: Unweighted Pair-Group Method with Arithmetic Mean; YL: Youli pig breed.

## Authors' contributions

All carried out research; MPE conceived research, analyzed data and wrote the manuscript; MLB supervised dissection and co-wrote the manuscript. All authors read and approved the final manuscript.

## Supplementary Material

Additional file 1 **Tissues sampled.** Click here for file

Additional file 2 **Complete list of sex z-scores for every tissue.** HYPO: Hypothalamus; AHYP: adenohypophysis; THYG: thyroid gland; GONA: gonads; FATB: backfat. Yellow underlined probesets correspond to the 100 most sex biased in each tissue. Click here for file

Additional file 3 **Over represented Gene Ontology (GO) categories among the 1700 most sex biased genes in gonads.** Click here for file

Additional file 4 **Means (SD) of the standard deviations of breed Bayesian z-scores.** Click here for file

Additional file 5 **Complete list of breed z-scores in each tissue.** LW: Large White; DU, Duroc; YL, Youli; IB, Iberian; HYPO: Hypothalamus; AHYP: adenohypophysis; THYG: thyroid gland; GONA: gonads; FATB: backfat; SD, standard deviation of breed z-scores for each breed and tissue. Click here for file
