# Supplementary material for: Impact of breed and sex on porcine endocrine transcriptome: a bayesian biometrical analysis
Source: BMC Genomics. 2009 Feb 24;10:89. doi: 10.1186/1471-2164-10-89 (PMC2656523; doi:10.1186/1471-2164-10-89)
Supplement: Additional file 1 — Tissues sampled. [file 1471-2164-10-89-S1.doc]

**Additional File 1.- Tissues sampled.**

| **Tissue** | **Abbreviation** | **Hormones secreted, general endocrine role** | **Sampling details** |
| --- | --- | --- | --- |
| Hypothalamus | HYPO | Release and release-inhibiting hormones (control AHYP), CRH, TRH, GHRH, SS, GnRH, Dopamine, MSH-IH | Including mammilary body and grey tubercle |
| Adenohypophysis  (anterior pituitary) | AHYP | Prolactin, growth hormone, MSH, ACTH, FSH, LH, ... | Whole gland, separated from neurohypophysis |
| Thyroid gland | THYG | Thyroid hormones (control metabolism level), calcitonin | Part of the gland |
| Gonad | GONA | Estrogens, progesterone, testosterone | Part of the organ |
| Back fat tissue | FATB | Leptin (targets hypothalamus) | Fat from lumbar region |
