# Supplementary material for: Impact of breed and sex on porcine endocrine transcriptome: a bayesian biometrical analysis
Source: BMC Genomics. 2009 Feb 24;10:89. doi: 10.1186/1471-2164-10-89 (PMC2656523; doi:10.1186/1471-2164-10-89)
Supplement: Additional file 3 — Over represented Gene Ontology (GO) categories among the 1700 most sex biased genes in gonads. [file 1471-2164-10-89-S3.doc]

**Additional File 3 : Over represented Gene Ontology (GO) category in the 1700 most sex biased genes in gonads**

| **GO ID** | **GO Name** | **P-Value** | **FDR** |
| --- | --- | --- | --- |
| GO:0007275 | multicellular organismal development | 2×10^-05^ | 0.04 |
| GO:0007155 | cell adhesion | 3×10^-04^ | 0.28 |
| GO:0007584 | response to nutrient | 4×10^-04^ | 0.28 |
| GO:0030199 | collagen fibril organization | 0.001 | 0.60 |
| GO:0008015 | circulation | 0.001 | 0.60 |
| GO:0007283 | spermatogenesis | 0.002 | 0.60 |
| GO:0006412 | translation | 0.003 | 0.66 |
| GO:0006512 | ubiquitin cycle | 0.005 | 0.66 |
| GO:0007595 | lactation | 0.005 | 0.66 |
| GO:0006397 | mRNA processing | 0.005 | 0.66 |
| GO:0000165 | MAPKKK cascade | 0.007 | 0.66 |
| GO:0007010 | cytoskeleton organization & biogenesis | 0.007 | 0.66 |
| GO:0008584 | male gonad development | 0.007 | 0.66 |
| GO:0030154 | cell differentiation | 0.007 | 0.66 |

FDR: False discovery rate
