# Supplementary material for: Impact of breed and sex on porcine endocrine transcriptome: a bayesian biometrical analysis
Source: BMC Genomics. 2009 Feb 24;10:89. doi: 10.1186/1471-2164-10-89 (PMC2656523; doi:10.1186/1471-2164-10-89)
Supplement: Additional file 4 — Means (SD) of the standard deviations of breed Bayesian z-scores. [file 1471-2164-10-89-S4.doc]

**Additional File 4. Means (SD) of the standard deviations of breed Bayesian z-scores .**

|  | Tissue | | | | | | | |
| --- | --- | --- | --- | --- | --- | --- | --- | --- |
| Probes | All | HYPO | AHYP | THYG | GONA | GONAM | GONAF | FATB |
| All | 0.82 (0.76) | 0.25 (0.32) | 0.38 (0.45) | 0.41 (0.48) | 1.86 (2.12) | 0.56 (0.63) | 0.10 (0.10) | 0.43 (0.48) |
| Largest 100 | 6.27 (2.14) | 2.34 (0.39) | 3.38 (0.62) | 3.57 (0.68) | 13.56 (1.16) | 4.92 (1.00) | 0.60 (0.03) | 3.47 (0.47) |
